# Small-sized newborn dogs skeletal development: radiologic, morphometric, and histological findings obtained from spontaneously dead animals

**DOI:** 10.1186/s12917-017-1092-6

**Published:** 2017-06-14

**Authors:** S. C. Modina, M.C. Veronesi, M. Moioli, T. Meloni, G. Lodi, V. Bronzo, M. Di Giancamillo

**Affiliations:** 1 Department of Health, Animal Science and Food Safety, Università degli Studi di Milano, Via Celoria 10 - 20133, Milano, Italy; 2 Department of Veterinary Medicine, Università degli Studi di Milano, Via Celoria 10 - 20133, Milan, Italy

**Keywords:** Dog, Small-sized breed, Newborn, Skeletal development, Morphometry, Radiography, Bone mineral density, Histology

## Abstract

**Background:**

Very little is known about neonatal skeletal development in small-sized purebred dogs. In order to improve this knowledge, 27 spontaneously dead puppies belonging to small-sized breeds were enrolled in this study for radiologic, histological and morphometric investigations.

**Results:**

The appearance of the limb secondary ossification centers and the onset of their formation were clearly observed by x rays and confirmed by histological evidences. Radiographic and anatomic measurements of limb bones length and skull length and width were positively correlated with body weight and age of the subjects and the body weight was positively correlated with radius bone mineral density, as demonstrated by dual-energy x-rays absorptiometry.

**Conclusions:**

These data provided original information on the growth of newborn small-sized breed dogs, and suggest that cadavers may be useful to study skeletal development.

**Electronic supplementary material:**

The online version of this article (doi:10.1186/s12917-017-1092-6) contains supplementary material, which is available to authorized users.

## Background

The skeletal development of a puppy entails deep changes in body size and shape and several factors can affect the modalities and timelines of post-natal growth and proportioning [1]. In the dog the wide variation in body size among breeds could be responsible for breed-specific differences in growth patterns [2, 3]. Currently, radiographic evaluation of the appearance and development of ossification centers (OCs) of limb bones is the most reliable method to estimate skeletal maturity in the dog [4–6]. However, the skeletal characteristics of growing dogs were also investigated by morphometric [7–9] and mineralometric approaches [10–12]. These data mainly refer to large and medium-sized breeds and, with regards to mineral density, to juvenile animals. Currently, the pattern of skeletal grows in newborn dogs up to one month of age is poorly described but deserves scientific interest. In fact from birth to the age of about 4 weeks, the newborn puppy ability to stand and walk evolves, from the initial crawling to the active well-coordinated walking. This happens thanks to the dynamic progress of body growth, coupled to simultaneous musculoskeletal and nervous systems maturation [13]. Unfortunately, studying the skeleton often requires invasive and repeated investigations, such as from x-rays and/or histological analysis [14, 15]. Moreover, a large and homogeneous sample is necessary to study skeletal development, and such condition is only guaranteed by experimental animals [6], a very expensive, time consuming and ethically questionable approach. Therefore, studies on skeletal development in dogs are often performed on animals belonging to several breeds, only grouped by known age [16].

A suitable compromise to the above-mentioned limits, could be the study of skeletal development in purebred newborn puppies, spontaneously and sudden dead, at well-known age, belonging to breeds categorized according to the body size. Moreover, cadaver is suitable not only for gross anatomy morphometric studies, as in living animals, but also for radiology procedures, as previously demonstrated [17]. The combined investigations could be useful for a more accurate measurement in the very small newborn dogs.

The aim of the present study was to investigate the morphometric and morphologic skeletal characteristics in small-sized purebred puppies during the first month of age. The investigation was conducted on spontaneously and sudden dead newborn dogs.

More specifically the study evaluated: the radiographic and histological appearance of the ossification centers (OCs) in fore- and hind-limb long bones; the relationship between long limb bones and skull measures obtained by both radiographic and anatomic approaches, and age and body weight; the relationship between *radius* Bone Mineral Density (BMD), age and body weight, and radiographic and anatomic *radius* measurements.

## Methods

### Animals

Twenty seven puppies, belonging to breeds categorized as small-sized according to the standard breed adult body weight < 7 kg [18], spontaneously dead at the age up to 28 days were enrolled in our Veterinary Teaching Hospital for educational purpose. They were supplied by owners (breeders) prior written informed consent, according to current Italian law.

All puppies were born full term, by healthy bitches, regularly vaccinated and dewormed before mating, with normal gestation, parturition and post partum course. During the second half of gestation, all the bitches were fed with pregnancy-specific commercial food. After death, puppies were stored at 4 °C for less than 12 h and refrigerated during the transfer to the Università degli Studi di Milano. Breed, gender, age (days after birth), and body weight were recorded for each cadaver before storage by freezing at −80 °C. According to the breed, the 27 puppies were distributed as follows: Chihuahua (*n* = 12), Maltese (*n* = 7), miniature Pinscher (*n* = 2), Shih-tzu (*n* = 3), toy Poodle (*n* = 3). Concerning to the gender, 12 puppies were females and 15 males, while according to the age at the time of death, four groups were identified: group I (puppies dead from birth to 7 days of age, *n* = 19), group II (puppies dead between 8 and 14 days of age, *n* = 4), group III (puppies dead between 15 and 21 days of age, *n* = 2) and group IV (puppies dead between 22 and 28 days of age, *n* = 2).

The cadavers were then thawed at room temperature and submitted to further investigations. All cadavers were submitted to the radiographic investigation. Thirteen subjects, including 5/19 puppies belonging to the first group of age, randomly chosen, and all the puppies belonging to the other three groups of age, were selected for further densitometric, anatomic and histological analyses.

### Radiographic examination and bone mineral density assessment

Radiographic images were acquired with a FCR Capsula X system (Fujifilm Italy S.p.A.) assembled with a radiological unit (Simply - Arcom S.r.l. Italy) using a 0.6 mm focal spot. The focal spot-film distance was 100 cm. For each puppy, forelimb and hindlimb ossification centers (OCs) were evaluated on medio-lateral (ML) and cranio-caudal (Cr-Cd) views. Hip was appreciated in ventro-dorsal view. For the morphometric measurements, right lateral (RL) and dorso-ventral (DV) views of the head were obtained. The OCs detection was identified as a radiopaque area on radiographs at the level of the future corresponding bone [19].

Bone mineral density (BMD) (g/cm^2^) was subsequently assessed. A medio-lateral scan was performed on the left radius by means of a dual-energy x-ray absorptiometry (DXA) device (Hologic QDR-1000 Plus, Hologic, Waltham, MA, USA). The radial shaft was divided in three areas of equal dimension, and the region of interest (ROI) was selected on the central part for each shaft including the full outline of the bone.

Before each scan, the bone densitometer was regularly submitted to quality control procedures with a specific phantom (Hologica Calibration Phantom, Hologic).

### Morphometry

Morphometry was evaluated by radiographic and anatomic measurements; every measurement was taken three times by a single operator in order to improve precision. The following radiographic measurements, performed by OsiriX^PRO^ 64-bit certified software (Aycan Medical Systems, LLC, Rochester, NY): skull length (SL) - from the external occipital protuberance to the anterior end of the inter-incisive suture; cranial length (CL) - from the junction of the median plane of the right and left naso-frontal sutures to the external occipital protuberance; neurocranium widths (NW) - from the most lateral point of the skull to the one of the other side; zygomatic widths (ZW) - from the most lateral point of one zygomatic arch to the most lateral point of the other [20, 21]. *Humerus* (HL), *radius* (RL) and *ulna* (UL), *os femoris* (FL)*, tibia* (TL) - a parallel line to the longest axis of the bone was drawn and the maximum length of the ossified bone was measured along this line. Since at birth only the diaphysis is radiopaque [22] radiographic long bones length corresponds with the length of the actual diaphysis [23].

Thirteen cadavers were submitted to gross anatomic measurements, performed by a traditional calliper (accuracy 5 mm). The *humerus, radius* and *ulna*, *os femoris* and *tibia* lengths were measured as the distance between the most proximal and distal points of the bone [24]. The latter was measured using palpable skeletal landmarks [25]. Skull length and NW were also measured [26].

In order to minimize the possible confounding effect of the surrounding soft tissues, a subsequent measurement of the long bones length was also performed after skeletonization of the limbs.

The terminology adopted was chosen in accordance to the *Nomina Anatomica Veterinaria* (2012).

### Histological investigation

**Fig. 1:**
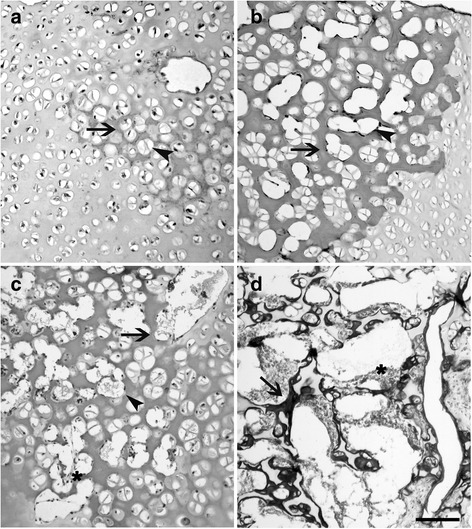
Representative histological images of an OCT-0 (**a**), an OCT-1 (**b**), an OCT-2 (**c**) and an OCT-3 (**d**). According with the progression of age, the enlargement of the lacunae (**a**, **b**, **c**, head arrow) and the thickening and calcification of the extra cellular matrix (**a**, **b**, **c**, arrows) indicate the appearance of an ossification centre. In OCT-2, primary *trabeculae* delimit *lacunae* containing bone marrows cells (**c**, **d**, asterisk). In OCT-3, neoformed bone tissue appears, laying on the *trabeculae* (**d**, arrow). **a**, **b**, **c** stained with haematoxylin-eosin; D stained with a Masson’s Trichrome stain. Bar 0.01 mm

Histological investigations were performed on the same 13 selected cadavers. The epiphyses of humerus and radius-ulna, os femoris and *tibia-fibula* were fixed in buffered 10% formalin (Bio-Optica, Milan, Italy), decalcified with 45% formic acid (Sigma Chemical Company, St. Louis, USA), for 2–3 days and then with 15% 0.5 M EDTA solution (pH 8.0 - Sigma Chemical Company) for 7–10 days [27] with slight modifications. *Tarsus, carpus* and *pelvis* were fixed in toto*.* Samples were dehydrated and embedded in paraffin. Serial sections were mounted on glass slides previously treated with Vectabond (Vector Laboratories, Burlingame, CA, USA) and stained with haematoxylin-eosin and Masson’s Trichrome Staining (Bio-Optica). The chronologic appearance and morphologic characteristics of the ossification centers (OCs) were evaluated according to Rivas and Shapiro [28]. On the base of their structural organization, OCs were classified as OCs type 1 (OCT-1), 2 (OCT-2) and 3 (OCT-3) (Table 1). Ossification centers type 1 were characterized by the presence of hypertrophic chondrocytes and early formation of *trabeculae* surrounded by a growth plate; in OCT-2 an increase of *trabeculae* between the hypertrophic chondrocytes and the appearance of bone marrows cells was observed; eventually, OCT-3 showed a network of primary and secondary *trabeculae*, surrounding enlarged and irregular spaces, containing large amount of bone marrow cells and neo-formed bone tissue as demonstrated by Trichrome staining. Pre-ossification/precocious ossification features, comparable to OCT-1, but not detected by x-rays were classified as OCT-0 (Fig. 1).

**Table 1:** Timing of fore limb and hind limb OCs appearance by histological investigation

Ossification centers	I group (n =5)	II group (n = 4)	III group (n = 2)	IV group (n = 2)
*Caput humeri*	OCT-0 (2)	OCT-0 (2) OCT-2 (2)	OCT-2 (1) OCT-3 (1)	OCT-3 (2)
*Trochlea humeri*			OCT-0 (1)	OCT-3 (2)
*Capitulum humeri*			OCT-1 (1)	OCT-0 (2)
*Caput radii*				OCT-2 (2)
*Trochlea radii*				OCT-2 (2)
*Os carpi intermedium*				OCT-2 (2)
*Os carpi radiale*				OCT-2 (1)
*Os carpi ulnare*				OCT-1 (2)
*Os carpi accessorium*		OCT-1 (1)	OCT-2 (1)	OCT-3 (2)
*Os pubis*	OCT-2 (3) OCT-0 (1)	OCT-0 (1) OCT-3 (3)	OCT-3 (2)	OCT-3 (2)
*Os carpi II*. *III*. *IV*				OCT-1/2 (1)
*Caput ossis femoris*		OCT-0 (3)	OCT-1 (1) OCT-2 (1)	OCT-3 (2)
*Trochlea*			OCT-0 (2)	OCT-3 (2)
*Condylus medialis femoris*				OCT-3 (1) OCT-0 (1)
*Condylus lateralis femoris*				OCT-3 (1) OCT-0 (1)
*Tibial proximal epiphysis*				OCT-3 (2)
*Cochlea tibiae*				OCT-2 (2)
*Calcaneus*	OCT-3 (5)	OCT-3 (4)	OCT-3 (2)	OCT-3 (2)
*Talus*	OCT-3 (4) OCT-0 (1)	OCT-3 (2)	OCT-3 (2)	OCT-3 (2)
*Os tarsi centrale*				OCT-3 (2)

### Statistical analysis

Statistical analysis was performed with the IBM SPSS Statistics 22.0 (IBM SPSS Inc., Armonk, USA). Normal data distribution of all the parameters was verified by Shapiro-Wilk test. Since data were not normally distributed the repeatability of three times measurement was assessed by a non-parametric Friedman test and median value of all parameters were used for correlation assessment by a Spearman bivariate test. The following correlations were assessed: 1) between all the radiographic measurement and all the other radiographic measurements, the increasing of age and weight of the subjects; 2) between anatomic lengths of *humerus*, *radius*, *tibia* and *os femoris* measured before and after skeletonization of the limbs; 3) between all the anatomic measurements and all the other measurements, the increasing of age and weight of the subjects; 4) between radiographic HL, RL, UL, FL, TL, SL and NW, and the corresponding gross anatomic measurements; 5) between BMD and radiographic and anatomic *radius* lengths, and increasing of age and body weight of the subjects. Significance was set at *p* < 0.05.

## Results

All the 27 puppies resulted of normal body weight in relation to the breed and the age at the time of death [2]. No physical abnormalities were detected.

### Radiographic and histological findings

**Fig. 2:**
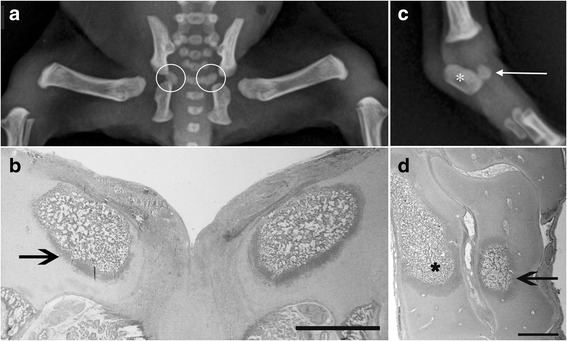
Radiographic ventro-dorsal view of the hip (**a**) and the corresponding histological section (**b**) of a 3 days-old Maltese dog: note the OCs for *os pubis* (white circles) that are both OCT-3 (**b** - black arrow); radiographic medio-lateral view of the *tarsus* (**c**) and corresponding histological section (**d**) of the same subject: OCs for *calcaneus* (asterisks) and *talus* (arrows) are clearly recognizable and are both OCT-3. Histological sections are stained with haematoxylin-eosin. Bar 1 mm

**Fig. 3:**
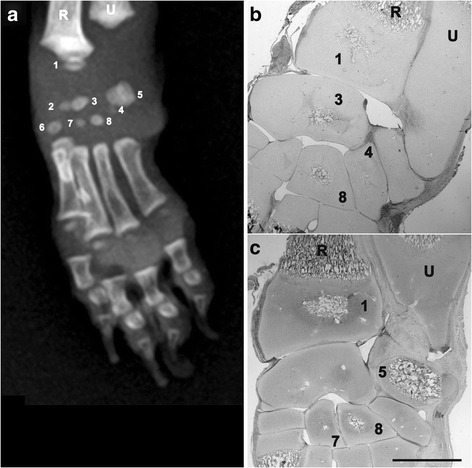
Twenty-eight days-old Chihuahua dog. Radiographic dorso-palmar view (**a**) and histological sections (**b**,**c**) of the left *carpus*: ossification centre of *trochlea radii* (1, OCT-2), *os carpi radiale* (2), *os carpi intermedium* (3, OCT-2), *os carpi ulnare* (4, OCT-1), the body of os carpi *accessorium* (5, OCT-3), *os carpi II* (6, OCT-1), *os carpi III* (7, OCT-1), *os carpi IV* (8, OCT-2). Different stage of development of the *carpus* OC’s are noticeable. Histological sections are stained with haematoxylin-eosin and are representatives of two different levels of depth; for this reason not all the nuclei are revealed. Bar 1 mm

**Fig. 4:**
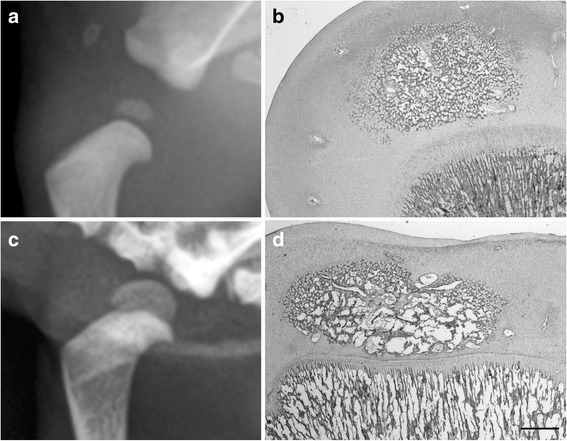
Radiographic medio-lateral views and the corresponding histological sections of the left *humerus* of a 15 days-old Maltese dog (**a**, **b**) and of a 28 days-old Chihuahua (**c**, **d**) respectively. With the progression of age, OC changes its aspect loosing the roughly round aspect (compare **a** and **c**), it flattens and passes from an OCT-2 (**b**) to an OCT-3 (**d**). Histological sections are stained with haematoxylin-eosin. Bar 0.5 mm

The body of the *scapula*, os ischi and os ilium and all the diaphysis of limb bones were already ossified at birth, while secondary long bones OCs and carpal bones OCs appeared later (Table 2). According to the progression of age, an increase of the ossification processes, coupled to a decrease in the relative amounts of cartilage mould, was detected by histology, confirming the radiographic findings. *Calcaneus, talus* and *os pubis* appeared at birth, but not in all subjects (Fig. 2). Carpal bones were detected only in the group IV of age (only one OC, with the exception of the *os carpi radiale* that showed two OCs encased in a unique cartilaginous mould) (Fig. 3). The *caput humeri* and the *caput ossis femoris* showed a roughly rounded OCs, in the group II and III, while a more flattened shape was observed in the group IV (Fig. 4).

**Table 2:** Percentage of fore limb and hind limb OCs radiographic appearance according to the groups of age

Ossification centers	I group (*n* = 19) 0–7 days	II group (*n* = 4) 8–14 days	III group (*n* = 2) 15–21 days	IV group (*n* = 2) 22–28 days
*Caput humeri*	0%	25%	100%	100%
*Trochlea humeri*	0%	0%	50%	100%
*Capitulum humeri*	0%	0%	50%	0%
*Caput radii*	0%	0%	0%	50%
*Trochlea radii*	0%	0%	0%	100%
*Os carpi radiale*	0%	0%	0%	50%
*Os carpi centrale*	0%	0%	0%	0%
*Os carpi intermedium*	0%	0%	0%	100%
*Os carpi accessorium*	0%	25%	50%	100%
*Os carpi ulnare*	0%	0%	0%	100%
*Os carpale II. III. IV*	0%	0%	0%	50%
*Os pubis*	31%	75%	100%	100%
*Caput ossis femoris*	0%	0%	50%	100%
*Trochlea femoris*	0%	0%	0%	50%
*Condylus medialis femoris*	0%	0%	0%	50%
*Condylus lateralis femoris*	0%	0%	0%	50%
*Cochlea tibiae*	0%	0%	0%	50%
*Calcaneus*	100%	75%	100%	100%
*Talus*	47%	100%	100%	100%
*Os tarsale I. II*	0%	0%	0%	0%
*Os tarsale III*	0%	0%	0%	50%
*Os tarsale IV*	0%	0%	50%	100%

### Morphometry

Long bones and skull radiographic measurements were positively correlated (*p* < 0.01), and always positively correlated with body weight (*p* < 0.01) and age (*p* < 0.05 and *p* < 0.01) (Table 3).

**Table 3:** Spearman’s correlation between age, body weight and radiographic measurements (*n* = 27)

	Age	Body weight	SL	NW	ZW	HL	RL	UL	FL	TL
Age	1.000	.453^*^	.676^**^	.474^*^	.805^**^	.457^*^	.484^*^	.570^**^	.503^**^	.582^**^
Body weight	.453^*^	1.000	.711^**^	.673^**^	.731^**^	.576^**^	.727^**^	.731^**^	.669^**^	.734^**^
SL	.676^**^	.711^**^	1.000	.596^**^	.852^**^	.644^**^	.708^**^	.763^**^	.742^**^	.820^**^
NW	.474^*^	.673^**^	.596^**^	1.000	.607^**^	.618^**^	.697^**^	.699^**^	.738^**^	.708^**^
ZW	.805^**^	.731^**^	.852^**^	.607^**^	1.000	.613^**^	.674^**^	.733^**^	.650^**^	.741^**^
HL	.457^*^	.576^**^	.644^**^	.618^**^	.613^**^	1.000	.923^**^	.877^**^	.904^**^	.820^**^
RL	.484^*^	.727^**^	.708^**^	.697^**^	.674^**^	.923^**^	1.000	.959^**^	.905^**^	.930^**^
UL	.570^**^	.731^**^	.763^**^	.699^**^	.733^**^	.877^**^	.959^**^	1.000	.891^**^	.942^**^
FL	.503^**^	.669^**^	.742^**^	.738^**^	.650^**^	.904^**^	.905^**^	.891^**^	1.000	.911^**^
TL	.582^**^	.734^**^	.820^**^	.708^**^	.741^**^	.820^**^	.930^**^	.942^**^	.911^**^	1.000

**p < 0.01; *p < 0.05

Anatomic morphometry of long bones showed that *humerus, radius* and *ulna*, *os femoris* and *tibia* lengths, recorded before and after skeletonization, were all positively correlated (*p* < 0.01 – data not shown).

Similarly to the radiographic findings, anatomic measurements were also positively correlated (*p* < 0.01 and *p* < 0.05) and always positively correlated with body weight (*p* < 0.01 and *p* < 0.05). Only SL and NW were positively correlated with the increasing age (*p* < 0.01) (Table 4).

**Table 4:** Spearman’s correlation between age, body weight and anatomic measurements (*n* = 13)

	Age	Body weight	SL	NW	HL	RL	FL	TL
Age	1.000	.676^*^	.793^**^	.828^**^	.319	.303	.508	.546
Body weight	.676^*^	1.000	.853^**^	.890^**^	.724^**^	.577^*^	.830^**^	.857^**^
SL	.793^**^	.853^**^	1.000	.872^**^	.624^*^	.677^*^	.732^**^	.762^**^
NW	.828^**^	.890^**^	.872^**^	1.000	.591^*^	.505	.769^**^	.709^**^
HL	.319	.724^**^	.624^*^	.591^*^	1.000	.773^**^	.872^**^	.732^**^
RL	.303	.577^*^	.677^*^	.505	.773^**^	1.000	.753^**^	.555^*^
FL	.508	.830^**^	.732^**^	.769^**^	.872^**^	.753^**^	1.000	.654^*^
TL	.546	.857^**^	.762^**^	.709^**^	.732^**^	.555^*^	.654^*^	1.000

**p < 0.01; *p < 0.05

When radiographic and anatomic measurements were concerned, a positive strong correlation between radiographic and anatomic measurements was found for SL, NW, FL and TL (*r* = 0.802; 0.907; 0.852; 0.824, respectively, *p* < 0.01), and for HL and RL (*r* = 0.635; 0.643, respectively, *p* < 0.05).

Bone mineral density of the *radius* increased with the age, and the statistical analysis showed strong positive correlation with the body weight (*r* = 0.852, *p* < 0.01), the radiologic length (*r* = 0.665, *p* < 0.05) and anatomic length (*r* = 0.698, *p* < 0.01) of the bone. No correlation with age was found.

Body weight, radiographic, anatomic and BMD measurements (expressed as means ± SD), according to the groups of age, are reported as descriptive statistics as (Additional file 1).

## Discussion

In the present study, some useful information about skeletal development during the first month of age, in small-sized purebred newborn dogs was provided.

Radiographic and histological findings showed that the timeline of fore- and hind-limb OCs appearance in newborn small-sized breeds was similar to what reported for medium and large-breeds dogs [5, 6], with the exception of the *os pubis*. In contrast to these breeds, in which the body of *os pubis* OC was already ossified at birth [29], in the small-sized breeds, the body of *os pubis* OC was detected only from the third week of age. This finding might be considered as a peculiar characteristic of small-sized dogs, even if a functional explanation of this finding is difficult. Moreover it suggests that in dog, the number, location and time of appearance and fusion of the ossification centers may not be species-specific, probably due to the variability between breeds. This is in contrast with previous works that indicated that ossification processes occur in the same manner within a species [30, 31]. Histology results largely confirmed radiological findings, but also allowed to appreciate the structural changes occurring during the development of OCs. According with Rivas and Shapiro, [28] a chronological classification of the OCs of the dog on the basis of their morphology was proposed. In the rabbit sixteen structural steps were identified [28], while in the dog only four steps were described, because of the reduced timeline of the samples. Ossification Centers Type 1, 2 and 3 matched with radiologic data: as radiopacity increased and enlarged, histology revealed a complex organization of *trabeculae* network (i.e. OCT-2), leading to the final shape of the epiphyseal bone (OCT-3). This confirmed what was previously demonstrated in growing Beagle dogs [32, 33]. Interestingly OCT-0 was identified only by histological analyses. This could be explained with a reduced amount of mineral salts in the extracellular matrix, insufficient to be detected by x-rays [34]. In fact, the first evidence of radio-opacity matched with the actual appearance of hypertrophic chondrocytes lying in the mineralized extra cellular matrix (i.e. OCT-1).

Based on its structure, OCT-0 could be compared to the pre-ossifications centers described in the cartilaginous trochlea of growing children with Magnetic Resonance Imaging and Computed Tomography, exploiting the presence of free water associated with hypertrophic chondrocytes [35]. This approach could be of interest in the study of skeletal development also in animals; however it needs to be evaluated in dogs.

The timing of ossification and growth rates of skeletal components have long been studied in several species, not only for single individual age definition, but also for assessing the normal prenatal growth rate, as demonstrated in humans [23] and pigs [36], and during the early postnatal period in mice [37]. In the present study the radiographic and anatomic measurements of limb bones and skull allowed to prove that OCs appearance and maturation are coupled with somatic growth in newborn dogs, irrespective of the breed.

In the present study, puppies belonging to some small-sized breeds were enrolled. Even if differences in body conformation are evident in adults of different breeds, the authors are not aware about similar studies on newborn dogs. Possible differences in skeletal conformation between the enrolled breeds were not evaluated in the present study, also because of the small number of subjects; for this same reason sex-related differences were not evaluated. Radiographic and anatomic measurements were positively correlated, suggesting that, as demonstrated on living adult dogs [25], the latter could be considered as a reliable, not harmful tool to assess the growth of a puppy. The positive correlation observed between measurements obtained before and after skeletonization further strengthened these results, demonstrating that in the newborn small-sized dogs, the thickness of the soft tissues does not affect the accuracy of the technique. In the present study, each measurement was repeated three times by the same operator, in order to improve precision. However, for the demonstration of reproducibility assessment, an inter-observer evaluation should have been performed.

In the last ten years, in human medicine, DXA became a standard procedure to evaluate skeletal growth through bone mineral content and BMD assessing, in preterm newborns [38], children and adolescents [39]. The results from the present study showed that the same procedure could be useful also for the growing dog. In fact, in this study, in newborn dogs bone mineralization changed during growth, similarly to bone structure, with an increase parallel to skeleton growth.

Previous studies in the dog, showed the usefulness of vertebral BMD to obtain reference bone density values in relation to age, gender and body weight in growing and young adult Boxer dogs [11], and the positive correlation between *femur* BMD and length with age [12] in juvenile large-breeds dogs. Similar results were also reported in juvenile rats and human *fetuses* [40, 41], suggesting that appendicular bones BMD might provide useful information in studies about growth in several species.

In this study the radius was chosen because it was more suitable for bone and anatomic region positioning and measuring, easily repeatable in all the subjects, even in a living animal. Moreover, in small-sized breeds, the achievement of precise information on radius might deserve particular interest also from a clinical perspective. These breeds in fact show a high predisposition to develop ante brachial fractures, as a consequence of falling or jumping [42] and the role of radial mineral accretion as predictor of bone failure is still under debated [43].

On the basis of previous studies, the radius BMD was expected to increase together with body weight, age and bone length. The results of this study showed a high positive correlation between BMD and body weight and a lower but still statistically significant correlation with bone length. Radius BMD in fact was not correlated with age. In the Authors’ opinion this may be caused by the relatively small number of animals enrolled in the present study. In addition, the interval of time ranging between birth and 28 days of age, could represent a too narrow window to assess the actual impact of age on bones development, and further investigations are needed using a proper size of studied animals during a longer observation time.

## Conclusions

Data from the present study allowed the achievement of important breed body-size specific characteristics on small-sized newborn dogs. The timeline and characteristics of OCs were defined; the positive correlation between radiographic and anatomic measurements of most of the studied parameters highlighted that gross anatomic measurements can be easily used to study newborn dogs; furthermore, the radius BMD, correlated to radiographic and anatomic measurements, proved to be useful for newborn dogs growing assessment. Finally, the present study highlighted the usefulness of spontaneously dead puppies for the radiologic, morphometric and histological investigations of skeletal development.

## Additional file

Additional file 1: Body weight, radiographic, anatomic and BMD measurements (expressed as mean ± SD) according to the group of ageDescription of data: Data about body weight, radiographic, anatomic and BMD measurements (expressed as mean ± SD) according to the group of age are reported (DOCX 13 kb).

## Abbreviations

OCs: Ossification centers
OCT-0: Ossification center type 0
OCT-1: Ossification center type 1
OCT-2: Ossification center type 2
OCT-3: Ossification center type 3
BMD: Bone Mineral Density
LL: Latero-lateral
DV: Dorso-ventral
ML: Medio-lateral
Cr-Cd: Cranio-caudal
DEXA: Dual-energy X-ray absorption
SL: Skull length
CL: Cranial length
NW: Neurocranium width
ZW: Zygomatic width
HL: Humerus length
RL: Radius length
UL: Ulna length
oFL: os Femoris length
TL: Tibia length
FL: Fibula length
