# Supplementary material for: Small-sized newborn dogs skeletal development: radiologic, morphometric, and histological findings obtained from spontaneously dead animals
Source: BMC Vet Res. 2017 Jun 14;13:175. doi: 10.1186/s12917-017-1092-6 (PMC5471892; doi:10.1186/s12917-017-1092-6)
Supplement: Additional file 1: — Body weight, radiographic, anatomic and BMD measurements (expressed as mean ± SD) according to the group of ageDescription of data: Data about body weight, radiographic, anatomic and BMD measurements (expressed as mean ± SD) according to the group of age are reported (DOCX 13 kb). [file 12917_2017_1092_MOESM1_ESM.docx]

Body weight, radiographic, anatomic and BMD measurements (expressed as mean±SD) according to the group of age

|  | Group 1 | | Group 2 | | | Group 3 | | | Group 4 | | |
| --- | --- | --- | --- | --- | --- | --- | --- | --- | --- | --- | --- |
|  | x-ray | anatomy | x-ray | anatomy | | x-ray | anatomy | | x-ray | anatomy | |
| n | 19 | 5 | 4 | | | 2 | | | 2 | | |
| BW | 111.6±31.39 | 119.6±23.45 | 109±20.99 | | | 150±21.21 | | | 265±21.21 | | |
| SL | 38±3.05 | 41.9±3.59 | 40.3±2.82 | | 42.6±2.69 | 42.8±0.45 | | 49±0.2 | 59±1.47 | | 62.9±1.4 |
| NW | 24.8±2.23 | 27.9±6.17 | 23.6±1.92 | | 28.4±3.1 | 31.1±1 | | 35.1±5 | 38.2±12.25 | | 43.6±4.6 |
| ZW | 24.1±2.81 |  | 27.9±2.12 | |  | 31.8±6.74 | |  | 40.1±1.2 | |  |
| HL | 15.8±1.65 | 24.4±1.6 | 15.8±1 | | 21.±2.17 | 16.4±0.26 | | 25.7±0.9 | 26.4±5,43 | | 35.7±1.2 |
| RL | 12.7±1.57 | 22.9±3.89 | 12.9±0.92 | | 19.2±1.64 | 13.8±0.24 | | 23.5±1.06 | 22.2±5.11 | | 36.7±1.67 |
| UL | 14.6±1.91 |  | 15.2±1.02 | |  | 16.3±0.14 | |  | 26.5±5.6 | |  |
| FL | 16±1.8 | 22.6±1.19 | 15.6±1.15 | | 20.9±1.85 | 17.6±0.59 | | 24.5±0.4 | 28.6±5.57 | | 33.2±2.76 |
| TL | 13.8±1.84 | 23.5 ±3.34 | 14.4±1.47 | | 20.3±0.96 | 15.5±0.47 | | 27.8±0.21 | 25.3±6.41 | | 35±2.1 |
| BMD |  | 0.12±0.04 | 0.08±0.03 | | | 0.14±0.02 | | | 0.19±0.02 | | |
